# Research on the Phenomenological Constitutive Relationship Model of Silicone Structural Adhesives for Glass Curtain Walls

**DOI:** 10.3390/polym17040547

**Published:** 2025-02-19

**Authors:** Nan Jin, Jianchao Zhao, Yuzhu Liang, Xiaoqing Zhao

**Affiliations:** 1 Shenzhen Key Laboratory of Urban Disasters Digital Twin, Shenzhen Technology Institute of Urban Public Safety, Shenzhen 518023, China; jinnan@szsti.org; 2 Safe Urban Development Institute of Science and Technology (Shenzhen), Shenzhen 518023, China; 3 Research Institute of Urbanization and Urban Safety, School of Future Cities, University of Science and Technology Beijing, Beijing 100083, China; m202110063@xs.ustb.edu.cn (J.Z.); m202420165@xs.ustb.edu.cn (Y.L.)

**Keywords:** silicone structural adhesives, constitutive relationships, hyperelasticity, mechanical testing

## Abstract

Silicone structural adhesives (SSAs) play a critical role in load transfer within glass curtain wall systems. With the increasing service life of existing glass curtain walls in recent years, their structural safety has become a significant concern across various societal sectors. Accurate characterization of the stress–strain relationship of SSAs is fundamental for evaluating the safety and performance of curtain wall structures. While most existing studies rely on hyperelastic constitutive models derived from rubber materials, employing parameter fitting to describe the constitutive behavior of SSAs, there remains a notable lack of research directly addressing constitutive models tailored specifically for SSAs. Although SSAs share similar chemical compositions with rubber after curing, their mechanical properties exhibit substantial differences. As a result, conventional hyperelastic constitutive models often fail to adequately capture the diverse mechanical responses of SSAs. To address this gap, this study begins with a comprehensive market survey to identify and select representative SSAs. Tensile and shear mechanical experiments are then conducted, with the stress–strain relationships during loading processes accurately captured using advanced techniques, such as Digital Image Correlation (DIC). Building on the Acoustic Emission (AE) algorithm, an intelligent algorithm is developed to optimize the fitting of hyperelastic constitutive parameters, enabling a critical evaluation of the applicability of existing phenomenological hyperelastic models to SSAs. Furthermore, in response to the limitations of current models, a novel and universally applicable constitutive model for SSAs is proposed. The robustness of this model is rigorously validated through comparative analysis with experimental data. The findings of this study provide a universal phenomenological constitutive model for SSAs, significantly enhancing the efficiency and accuracy of constitutive relationship fitting for these materials. This advancement contributes to the improved design, assessment, and maintenance of glass curtain wall systems.

## 1. Introduction

Glass curtain walls have become a ubiquitous feature in public buildings across medium-sized and large cities, including museums, airports, theaters, shopping malls, hotels, and office buildings. Statistical data indicate that the total area of existing glass curtain walls has exceeded 1 billion square meters [1], with a significant concentration in cities experiencing robust commercial activity. As the service life of existing glass curtain wall buildings increases, safety incidents have become more frequent. Currently, spontaneous breakage and detachment of glass curtain walls pose major safety hazards in urban development. Silicone structural adhesives (SSAs) is a crucial material for load transfer in glass curtain walls, and its mechanical properties directly influence the safety of these structures. Accurately characterizing the stress–strain relationship of SSAs is essential for assessing its safety.

**Figure 1:**
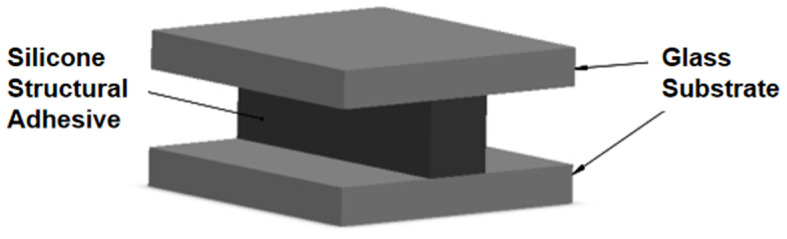
Schematic diagram of the specimen.

**Figure 2:**
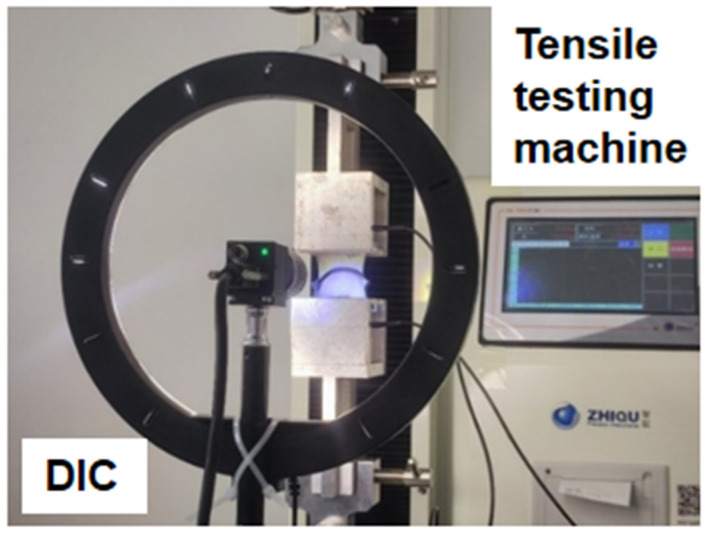
Schematic diagram of the loading and testing device.

**Figure 3:**
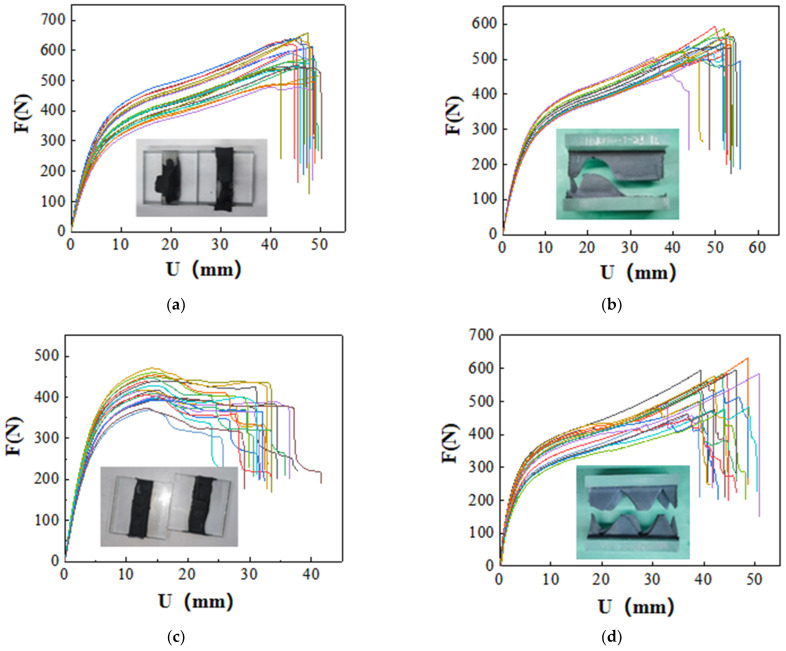

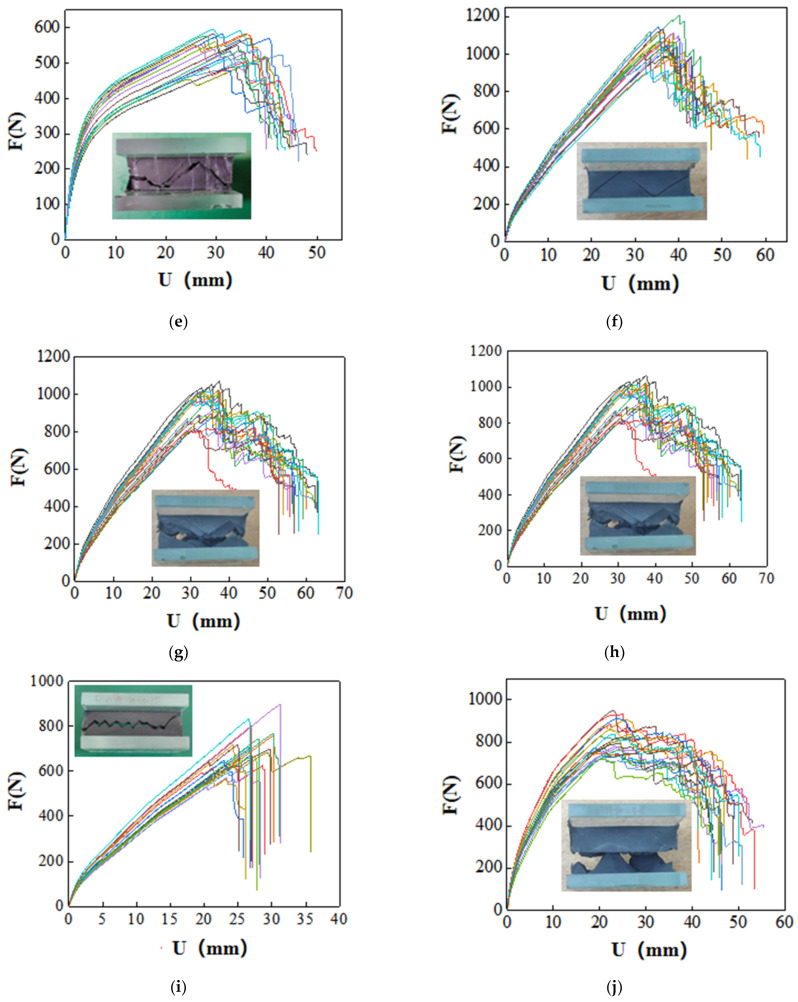
Summary of tensile test results for ten types of SSAs: (**a**) Tensile curve and typical cross-section of specimen A-1. (**b**) Tensile curve and typical cross-section of specimen A-2. (**c**) Tensile curve and typical cross-section of specimen B-1. (**d**) Tensile curve and typical cross-section of specimen B-2. (**e**) Tensile curve and typical cross-section of specimen C-1. (**f**) Tensile curve and typical cross-section of specimen D-1. (**g**) Tensile curve and typical cross-section of specimen D-2. (**h**) Tensile curve and typical cross-section of specimen E-1. (**i**) Tensile curve and typical cross-section of specimen E-2. (**j**) Tensile curve and typical cross-section of specimen F-1.

**Figure 4:**
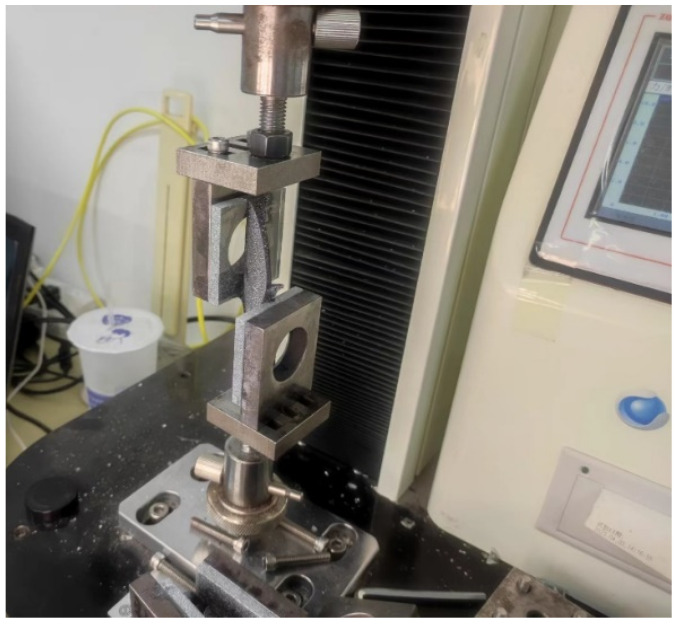
Loading device for the shear test.

**Figure 5:**
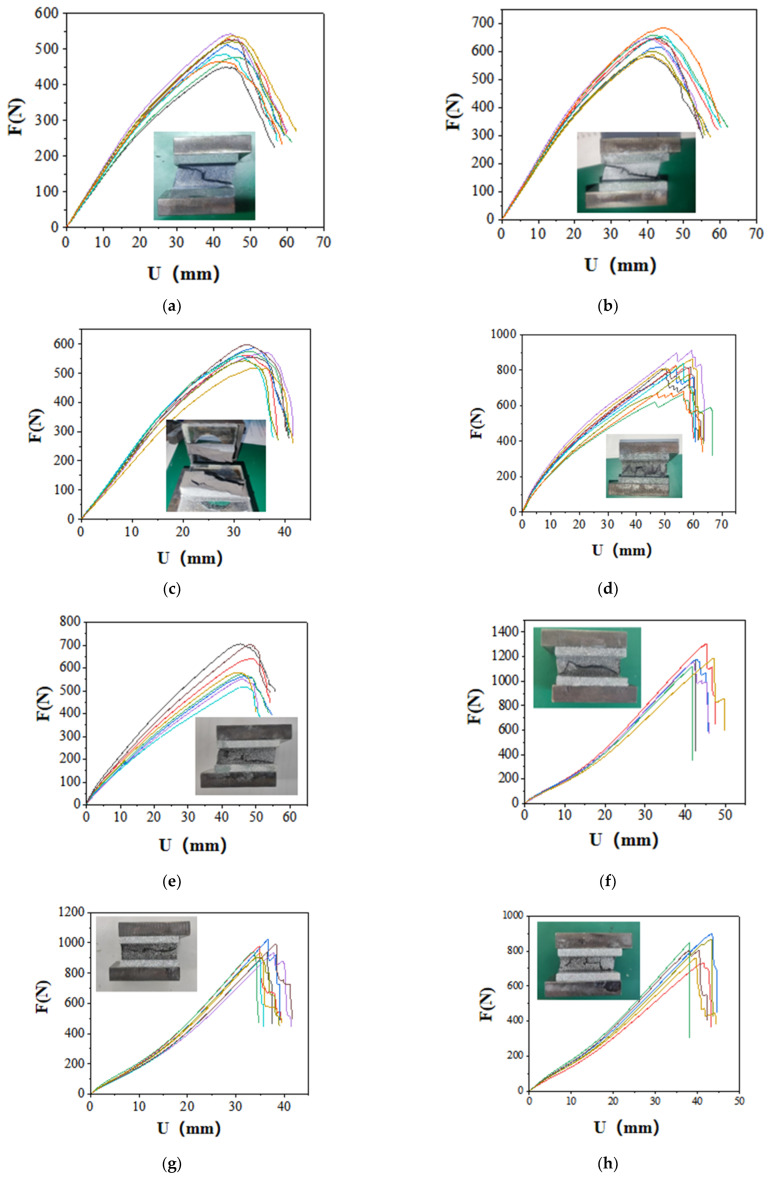

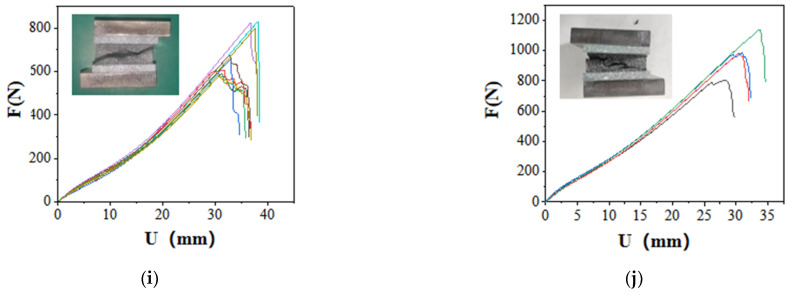
Summary of shear test results for ten types of SSAs: (**a**) Shear curve and typical cross-section of specimen A-1. (**b**) Shear curve and typical cross-section of specimen A-2. (**c**) Shear curve and typical cross-section of specimen B-1. (**d**) Shear curve and typical cross-section of specimen B-2. (**e**) Shear curve and typical cross-section of specimen C-1. (**f**) Shear curve and typical cross-section of specimen D-1. (**g**) Shear curve and typical cross-section of specimen D-2. (**h**) Shear curve and typical cross-section of specimen E-1. (**i**) Shear curve and typical cross-section of specimen E-2. (**j**) Shear curve and typical cross-section of specimen F-1.

**Figure 6:**
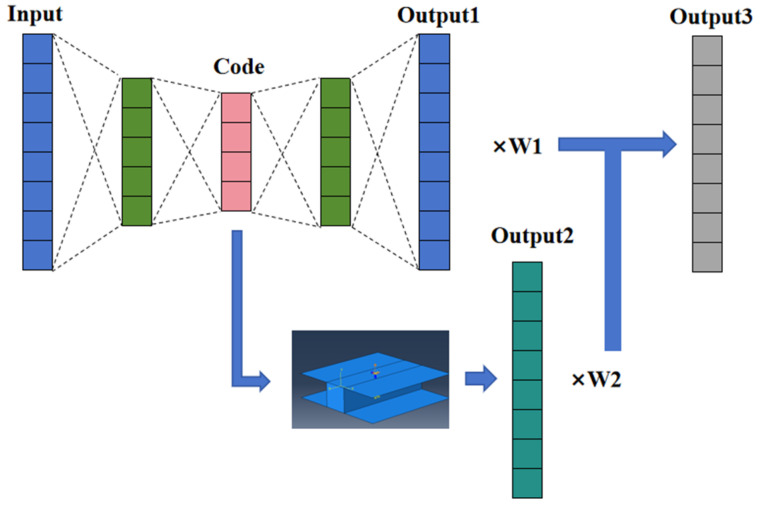
Hyperelastic constitutive relation parameter optimization algorithm based on the AE model.

**Figure 7:**
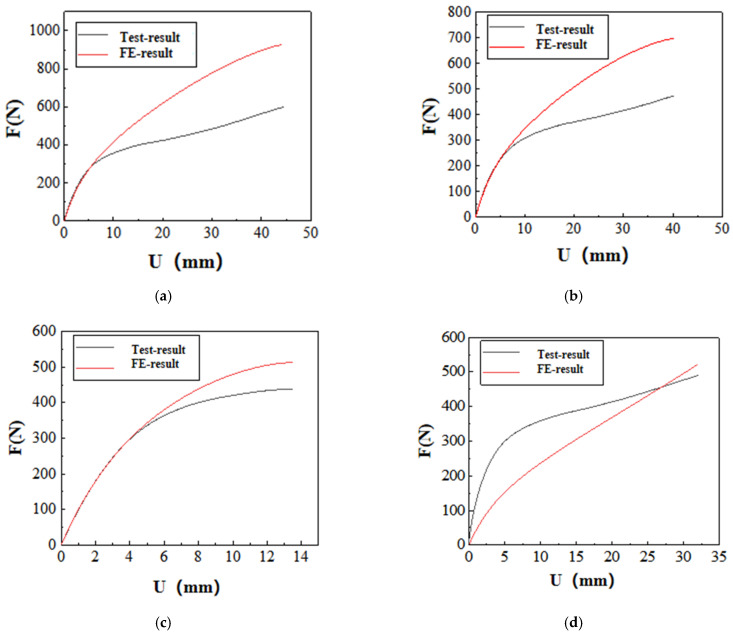
Load–displacement curve fitting for a reduced polynomial model with N = 2: (**a**) Comparison of load–displacement curves for specimen A-1. (**b**) Comparison of load–displacement curves for specimen A-2. (**c**) Comparison of load–displacement curves for specimen B-1. (**d**) Comparison of load–displacement curves for specimen B-2.

Currently, research on the mechanical properties of SSAs primarily focuses on their aging characteristics [2], with limited studies directly addressing their constitutive relationship models. After curing, the composition of SSAs closely resembles that of silicone rubber, and both materials exhibit certain similarities in mechanical properties, including large deformation, non-linearity, and hyperelasticity. As a result, the constitutive models developed for silicone rubber are often applied to describe the mechanical behavior of SSAs. When investigating the constitutive relationship of silicone rubber materials, they are typically considered as isotropic and incompressible hyperelastic bodies. Hyperelastic materials are defined as those that can return to their original shape after experiencing large strains, and their stress–strain relationship can be represented by a strain energy function. Numerous theoretical models have been proposed to describe hyperelastic mechanical behavior [3,4]. The theoretical hyperelastic constitutive models for silicone rubber can be broadly categorized into two types: those based on kinetic theory derived from statistical thermodynamics and those based on phenomenological theory rooted in continuum mechanics [5]. The former primarily examines the mechanical response of materials from a microstructural perspective, encompassing models such as the three-chain model [6], the eight-chain model [7], and the full-chain model [8]. In contrast, the latter does not take microstructure into account and characterizes the macroscopic stress–strain relationship through a mathematical framework, which mainly includes the polynomial model and the Ogden model [9], among others. Currently, phenomenological models are more widely applied to rubber materials. The essence of these models lies in establishing the expression for stored elastic energy, specifically the relationship defined by the strain–energy function. Commonly used phenomenological constitutive models are summarized in Table 1. In the polynomial model, when N = 1, it corresponds to the classic Mooney–Rivlin model [10]. Proposed in the 1940s, this model is the most widely used hyperelastic constitutive model and has significantly influenced the development of subsequent hyperelastic constitutive models. It accommodates changes in curve shape and is relatively effective for fitting small to medium deformations; however, it is less suitable for large deformations. In the reduced polynomial model, the influence of the second invariant is disregarded, which simplifies the formula and decreases the number of unknown parameters. Although the second invariant is omitted, the reduction in unknown parameters associated with it can, in some cases, lead to improved fitting results. When N = 1, it is a Neo-Hookean model [11], which is the simplest constitutive model. Although it has a straightforward formulation, its applicability is somewhat limited due to its reliance on a single parameter, making it primarily suitable for small-deformation cases. In contrast, the Yeoh model [12] is a third-order reduced polynomial model that offers a broader range of applications. This model can demonstrate the phenomenon of stiffness increase in rubber materials under large deformations, making it a commonly used constitutive model for scenarios involving significant deformation. The polynomial model represents the strain–energy function in terms of strain invariants, while the Ogden model [13] expresses the strain–energy function using principal stretches instead of invariants. This model demonstrates high fitting accuracy when sufficient data are available and can also exhibit the stiffening phenomenon of rubber materials under large strains. However, its primary drawback is its stringent requirements for data quality; complete test data are essential, making it less suitable when the data are relatively limited. Notably, when the coefficient is 2 and N = 2, the Ogden model is equivalent to the Mooney–Rivlin model. The Arruda–Boyce model [7] is a hyperelastic model developed based on a typical hexahedral unit. Its principle is that eight long chains radiate from the center to each vertex, which is why it is also referred to as the eight-chain model. This model is characterized as a two-parameter model, and its stability can be ensured as long as the parameters are positive values. However, due to having only two coefficients, its ability to alter the shape of the curve is limited. When the test data are restricted, the simulation performance of this model remains relatively good, and the two material parameters can modify the shape of the stress–strain curve. The specific formula is presented in Table 1, where μ primarily controls the initial segment of the curve, while λ_m_ governs the large-strain stiffening section of the strain curve. As illustrated in Figures 1–7, these are two typical fitting results of the Arruda–Boyce model, and it is not possible to achieve a good fit simultaneously in both the initial segment and the stiffening section. The Van der Waals model [14] is a four-parameter model, as detailed in Table 1. Depending on the specific parameters, the model can generate curves with varying functions and shapes. The parameter μ adjusts the curve vertically, while the parameter λ_m_ modifies it horizontally. The parameter α influences the overall shape of the curve, and the linear mixing parameter β regulates the relative shape changes under different deformation modes, allowing the model to be applicable to a wide range of experiments. When the experimental data are relatively uniform, it is advisable to set β = 0 to enhance fitting accuracy. These parameters contribute to the model’s superiority over the Arruda–Boyce model in terms of application range and accuracy of fitting results. However, this model requires high-quality experimental data. These models each have their own applicable ranges. In practical applications, it is essential to verify the suitability of the constitutive model using experimental data, which typically requires substantial computational effort. Although the components of SSAs are similar to those of rubber after curing, there are significant differences in their mechanical properties. Furthermore, existing hyperelastic constitutive models often struggle to accommodate the diverse range of SSAs.

Furthermore, existing research on SSAs primarily focuses on their aging properties, fatigue behavior, and dynamic effects. For instance, reference [15] simultaneously considers the aging and low-cycle fatigue effects of SSAs, proposing a novel hyperelastic constitutive model to simulate these behaviors. Reference [16] provides a systematic review of constitutive relationship studies for SSAs, particularly those incorporating failure stages. However, these studies predominantly rely on existing hyperelastic constitutive models as their foundation, without specifically addressing the applicability of these models to different types of SSAs. This limitation highlights the need for more targeted investigations into the suitability of existing models for the diverse mechanical behaviors exhibited by various SSAs.

In summary, there is currently a scarcity of literature directly investigating the constitutive relationship of SSAs. Most existing studies rely on hyperelastic constitutive models developed for rubber, focusing primarily on parameter fitting. This approach results in relatively low efficiency and accuracy when characterizing the stress–strain relationship of SSAs. To address this gap, this study first selects several representative SSAs through comprehensive market research. Tensile and shear mechanical experiments are then conducted, with the stress–strain relationships during loading accurately captured using advanced techniques such as Digital Image Correlation (DIC). Building on the Acoustic Emission (AE) algorithm, an intelligent algorithm is developed to optimize the fitting of hyperelastic constitutive parameters, enabling a critical evaluation of the applicability of existing phenomenological hyperelastic models to SSAs. Finally, in response to the limitations of current models, a universal constitutive model for SSAs is proposed. The robustness of this model is rigorously validated through comparative analysis with experimental data, providing a reliable framework for characterizing the mechanical behavior of SSAs.

## 2. Mechanical Testing of Typical SSAs

As a crucial component of glass curtain wall systems, SSAs play a vital role in ensuring structural safety and performance. The market offers a wide variety of silicone sealant products; however, their quality can vary significantly, leading to inconsistencies in mechanical properties across different types of SSAs. The quality of the silicone sealant directly affects the bond integrity between glass panels and the supporting structure, ultimately influencing the overall safety and durability of the glass curtain wall. To address this variability, this section presents the results of a comprehensive market survey, in which ten representative silicone structural sealants were selected for tensile and shear mechanical testing. The labels, application scopes, and key characteristics of these ten SSAs are summarized in Table 2.

### 2.1. Tensile Test

The specimens for the tensile test of silicone sealant were prepared following the recommended methods outlined in Part 8: Determination of Tensile Adhesion [17] of Test Methods for Building Sealants—2017 and Silicone Structural Sealants for Building—2005 [18]. The geometry and dimensions of the specimen are illustrated in Figure 1. The bonding materials used are SSAs, and the substrate is an ultra-white float glass. The dimensions of the substrate are 50 mm × 50 mm × 6 mm, while the dimensions of the sealant are 50 mm × 12 mm × 12 mm. Prior to use, the glass substrate is rinsed with alcohol, cleaned with purified water, and dried with lint-free paper. After the specimens are prepared, they are cured in a constant-temperature incubator for 21 days. The curing environment is maintained at a temperature of 23 °C and a humidity level of 50%. Twenty specimens are prepared for each type of SSAs. Once the specimens have cured, a 5 kN static testing machine is employed to conduct the tensile test. The loading speed is set at 5 mm/min, and the test is terminated when the load decreases to half of the maximum load. During the loading process, a Digital Image Correlation (DIC) device is utilized to record the stress–strain history of the test. The loading and testing apparatus is depicted in Figure 2.

The tensile test results for ten types of SSAs are summarized in Figure 3:

As illustrated in Figure 3, the tensile properties of the SSAs exhibit considerable variability, despite the fact that the sealant used to create the specimens was sourced from the same tube. For the Type A-1 and Type A-2 specimens, during the initial loading phase (0–10 mm), the load increases rapidly with displacement. In the intermediate loading phase (10–40 mm), the load rises more gradually, and the specimens demonstrate significant deformation capacity. In the final loading phase (40–50 mm), most specimens experience adhesive failure, characterized by the tearing of the colloid. This failure occurs at the bonding interface between the colloid and the substrate, alongside a simultaneous internal cohesion failure within the colloid itself. The failure process is notably abrupt, resulting in an “inverted S” shape on the tensile curve. Although there is a substantial difference in hardness values between the Type A-1 and Type A-2 SSAs, as shown in Figure 3a,b, the disparity in their tensile properties is not significant. Compared to Type A-1 and Type A-2 specimens, Type B-1 specimens exhibit a weaker ability to deform. During the initial loading stage (0–10 mm), the load increases rapidly as displacement increases. In the middle loading stage (10–15 mm), the load reaches its peak relatively quickly. In the later loading stage (15–40 mm), most specimens experience colloid tearing. The failure process demonstrates significant ductility, and there is no failure at the bonding interface between the colloid and the substrate. The failure mode observed in the specimens is cohesive failure of the colloid. The test results for Type B-2 specimens indicate considerable instability. For some specimens, the colloid suddenly fractures after the load reaches its peak, resulting in cohesive failure. For other specimens, after reaching the peak load, the colloid exhibits layered tearing. The failure process displays strong ductility characteristics, with a serrated cross-section shape and an “inverted S” phenomenon appearing on the tensile curve. After the load of Type C-1 specimens reaches its peak, the surface of the colloid gradually begins to peel off, resulting in a gradual decrease in load. This decrease occurs in a stepwise manner until the predetermined termination condition for the test is met. The cross-section of the specimen after failure exhibits a serrated appearance. In contrast, the test results for Type D-1 specimens demonstrate good stability. These specimens gradually fail after the load peaks, with the surface of the colloid peeling off layer by layer, leading to a gradual reduction in load until the termination condition is ultimately reached. The failure mode observed is a cohesive failure of the colloid. The test results for Type D-2 and Type E-1 specimens are quite similar; after reaching the peak load, the surface layer of the colloid peels off in layers, and the load decreases incrementally. The cross-section of the specimen exhibits a serrated texture with distinct layering, and the failure mode is characterized as cohesive failure. Type E-2 specimens fail rapidly and completely upon reaching the peak load. Throughout the loading process, the load consistently increases, and the slope of the curve remains relatively stable. Once the peak load is attained, the load experiences a sudden drop, leading to rapid damage of the specimen. The cross-section of the specimen is finely serrated, with no internal layering present within the adhesive layer. In contrast, after Type F-1 specimens reach their peak load, their bearing capacity gradually diminishes. The adhesive material exhibits a peeling phenomenon, detaching layer by layer from the interior. Following failure, the fracture displays a V-shape, and this type of failure is also classified as cohesive failure.

As indicated by the test results, the tensile failure modes of SSAs are predominantly cohesive failures, with only a few specimens exhibiting failure at the bonding interface between the sealant and the substrate. Analyzing the shape characteristics of the load–displacement history curves, Types A-1, A-2, and B-2 SSAs can be classified into the same category. During the initial loading phase, the load increases rapidly, transitions into a ductile stage, and ultimately fails suddenly, displaying an “inverted S” shape on the tensile curve. In contrast, for Type B-1 SSAs, the load also increases rapidly at the beginning of the loading process but is followed by a relatively extended descending section. Compared to Type A-1, A-2, and B-2 SSAs, the load–displacement curve of Type C-1 SSAs does not exhibit the characteristic “inverted S” shape. The load–displacement curves of Types D-1, D-2, E-1, and F-1 SSAs display similar linear characteristics. The load-rising section of Type E-2 SSAs resembles that of D-1, D-2, E-1, and F-1; however, the descending section is markedly abrupt. In summary, the load–displacement curves of the ten typical SSAs can be categorized into five distinct groups. By comparing this classification with the hardness values presented in Table 2, it becomes evident that the similarity in tensile properties among SSAs is not related to their hardness values. This further indicates that distinguishing the mechanical properties of SSAs based solely on hardness measurements is challenging.

### 2.2. Shear Test

Referring to the standard GB/T13477-2017 [17], shear performance tests were conducted on ten types of silicone adhesives. The selection of structural adhesive types, specimen preparation, and curing methods were consistent with those used in the tensile tests. A 5 kN static testing machine was employed for the tensile tests, with a loading speed set at 5 mm/min. The test was terminated when the load decreased to half of the maximum load. During the loading process, the Digital Image Correlation (DIC) equipment was utilized to record the stress–strain history of the tests. The loading and testing setup is illustrated in Figure 4.

The summary of the shear test results for ten types of SSAs is presented in Figure 5.

As illustrated in Figure 5, the load–displacement curves for the SSAs specimens of types A-1, A-2, B-1, B-2, and C-1 exhibit similar trends. However, there are notable differences in the tensile and shear failure modes of the specimens. During the shear test, the specimens do not experience sudden failure upon reaching the peak load. Instead, the material is gradually sheared until failure occurs. Upon failure, an inclined crack forms on the surface of the material, and no layer-by-layer peeling of the colloid is observed. There is no abrupt change at the peak load; rather, a gradual transition occurs. From the perspective of the failure process, as the displacement of the clamping end increases, the load continues to rise. After reaching the peak load, tearing begins to occur on the surface of the specimen, specifically at the interface between the colloid and the glass substrate. This tearing initiates at the edges of both ends and progresses inward. Subsequently, the cracks at both ends continue to expand until the upper and lower failure cross-sections intersect, leading to the final failure of the specimen. The load–displacement curves of the silicone structural sealant specimens—types D-1, D-2, E-1, E-2, and F-1—exhibit similar trends. These specimens experience sudden failure after reaching the peak load. When shear failure occurs, an inclined crack forms on the surface of the colloid, and no evidence of the colloid peeling off layer by layer is observed.

Combined with the hardness values presented in Table 2, it is evident that there is no significant correlation between the shear properties of the silicone sealant test and the hardness value of the colloid.

## 3. Applicability of Existing Phenomenological Constitutive Relations

### 3.1. Hyperelastic Constitutive Relationship Parameter Optimization Algorithm Based on the AE Algorithm

The optimization of parameters in traditional hyperelastic constitutive relations necessitates experimental data obtained under specific conditions, such as uniaxial compression, uniaxial tension, plane compression, plane tension, equi-biaxial compression, equi-biaxial tension, and volume compression. These experiments impose particular requirements on loading devices and testing equipment, which limits their widespread application in practical engineering. Consequently, references [19,20] proposed an inverse algorithm for determining hyperelastic constitutive relation parameters by integrating the genetic algorithm with the K-nearest neighbor algorithm. This method utilizes the load–history curve of tensile specimens to establish an error function that compares the finite element calculation results with the experimental curve. It employs optimization algorithms or machine learning techniques to identify the hyperelastic constitutive parameters that minimize the error function. The principle underlying this method indicates that it is not constrained by the experimental form and possesses significant engineering application value. To further enhance optimization efficiency, this section proposes a hyperelastic constitutive relation parameter optimization method based on the AE model, building on the aforementioned approach. The algorithm’s calculation flow is illustrated in Figure 6.

As illustrated in Figure 6, the hyperelastic constitutive relation optimization algorithm described in this section utilizes the load–displacement curve of the specimen as input. This input is encoded through a fully connected layer, with the dimensionality of the code layer matching the number of parameters in the constitutive relation model to be fitted. Following the code layer, two branches are established. One branch is a normal decoding layer, which is responsible for reconstructing the data from the input layer. The other branch involves finite element analysis, which generates the load–history curve corresponding to the input data. Subsequently, output 1 and output 2 are multiplied by their respective weights and then combined to produce the final output, output 3. Output 3 represents a load–displacement curve that corresponds to the input layer. The error function is defined as the sum of the squares of the differences between output 3 and the input. When this error function is minimized, it indicates that the parameter optimization has achieved its optimal state.

### 3.2. Optimization of Constitutive Parameters for SSAs

Based on the parameter optimization algorithm outlined in Section 3.1 and utilizing the tensile test data presented in Section 2.1, the parameters of the hyperelastic constitutive relations listed in Table 1 were optimized. The results indicate that reproducing the tensile load–displacement history curves for the SSAs specimens of types A-1, A-2, B-1, B-2, and C-1 using existing phenomenological constitutive relation models is challenging. Among these types of SSAs, the reduced polynomial constitutive model with N = 2 demonstrates the best reproducibility. A comparison of the finite element calculated values of its optimal parameters with the experimental results is illustrated in Figure 7.

As illustrated in Figure 7, although the aforementioned parameter optimization results are optimal, the error during the gentle stage remains significant. Currently, for these four types of SSAs, the existing phenomenological hyperelastic constitutive models are only applicable during the initial loading phase.

**Figure 8:**
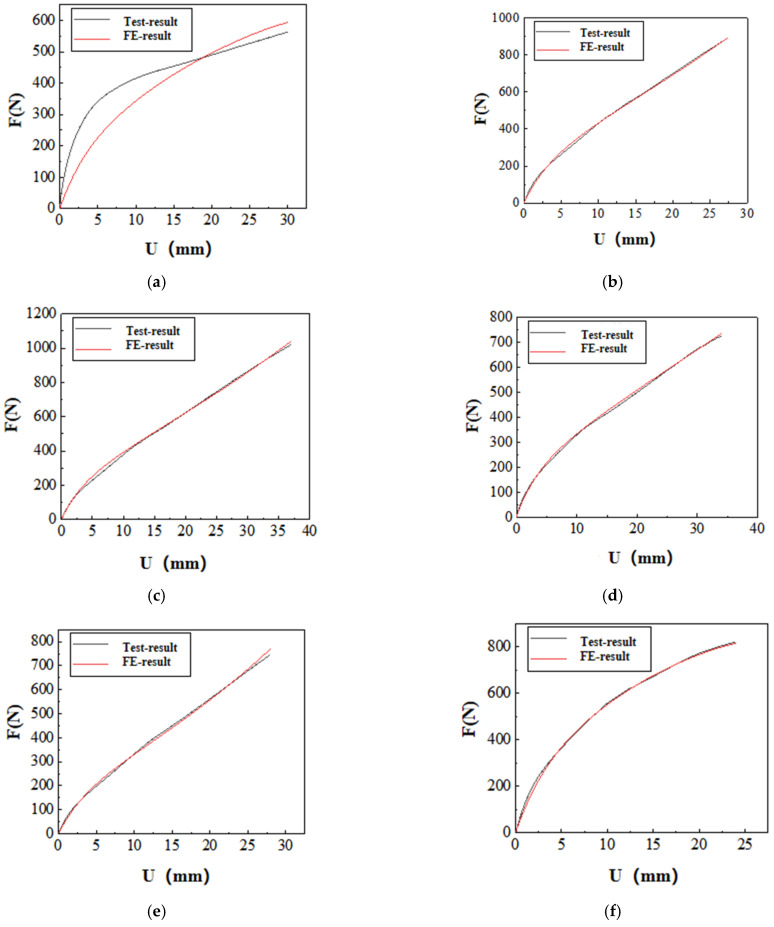
Load–displacement curve fitting for a reduced polynomial model with N = 2: (**a**) Comparison of load–displacement curves for specimen C-1. (**b**) Comparison of load–displacement curves for specimen D-1. (**c**) Comparison of load–displacement curves for specimen D-1. (**d**) Comparison of load–displacement curves for specimen E-1. (**e**) Comparison of load–displacement curves for specimen E-2. (**f**) Comparison of load–displacement curves for specimen F-1.

The inversion results of the constitutive parameters for the other types of SSAs specimens are presented in Figure 8.

As shown in Figure 8, the existing hyperelastic constitutive models effectively replicate the load–history curves of the SSAs specimens of types D-1, D-2, E-1, E-2, and F-1. The finite element analysis results closely align with the experimental data. However, the reproducibility of the load–history curve for the Type C-1 SSAs specimen is inadequate.

From the calculations and analysis presented above, it is evident that the intelligent algorithm for the inverse analysis of hyperelastic constitutive parameters, as proposed in Section 3.1, is capable of effectively performing inverse analysis on the constitutive parameters. Additionally, it can optimize these parameters based on the load–displacement curve. While existing hyperelastic constitutive models can characterize the stress–strain relationships of certain silicone structural sealants, they do not do so comprehensively.

## 4. Integral-Type Constitutive Relation Model

In order to develop a hyperelastic constitutive relation model that is universally applicable to SSAs, this paper compares various forms of existing hyperelastic constitutive relation strain energy functions. Through repeated calculations and derivations, it proposes a hyperelastic constitutive relation model in the following form:

(1) $$U=\int \left(C_{30}+\frac{C_{10}}{C_{20}+C_{60}(1-E^{C_{50}(I_{1}-3)})+{\left(I_{1}-3\right)}^{C_{40}}}\right)dI_{1}$$

(2) $$\frac{\partial U}{\partial I_{1}}=C_{30}+\frac{C_{10}}{C_{20}+C_{60}(1-E^{C_{50}(I_{1}-3)})+{\left(I_{1}-3\right)}^{C_{40}}}$$

where $C_{30}+\frac{C_{10}}{C_{20}}$ is the initial shear stiffness, $C_{30}$ is the tangent stiffness in the high-strain stage, $C_{40},C_{50},C_{60}$ are shape adjustment parameters of the strain–energy function, which are utilized to minimize errors.

The results of parameter inversion for the ten types of SSAs specimens mentioned above, using this integral-type constitutive relation model, are summarized in Table 3.

**Figure 9:**
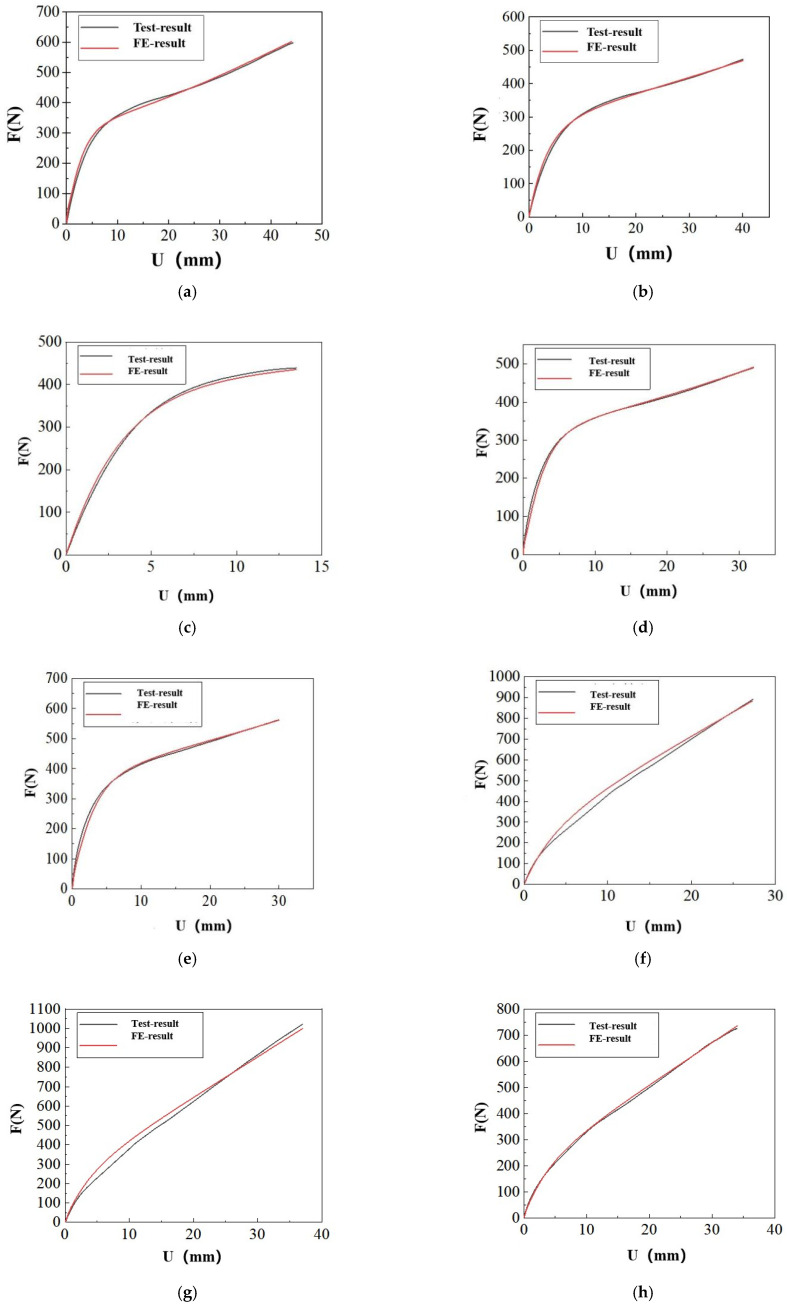

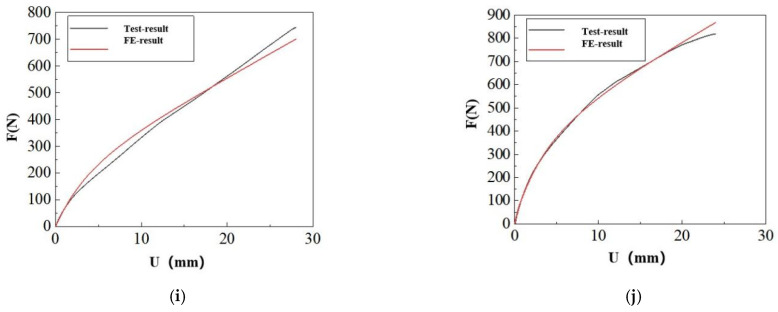
Load–displacement curve fitting for a reduced polynomial model with N = 2: (**a**) Comparison of load–displacement curves for specimen A-1. (**b**) Comparison of load–displacement curves for specimen A-2. (**c**) Comparison of load–displacement curves for specimen B-1. (**d**) Comparison of load–displacement curves for specimen B-2. (**e**) Comparison of load–displacement curves for specimen C-1. (**f**) Comparison of load–displacement curves for specimen D-1. (**g**) Comparison of load–displacement curves for specimen D-2. (**h**) Comparison of load–displacement curves for specimen E-1. (**i**) Comparison of load–displacement curves for specimen E-2. (**j**) Comparison of load–displacement curves for specimen F-1.

The comparison between the results of the finite element calculations and the experimental results is as follows:

As shown in Figure 9 and Table 3, the integral-type constitutive relation proposed in this section demonstrates strong universality across ten types of SSAs. The load–displacement curves derived from finite element calculations closely align with the experimental results.

## 5. Comparison and Verification

**Figure 10:**
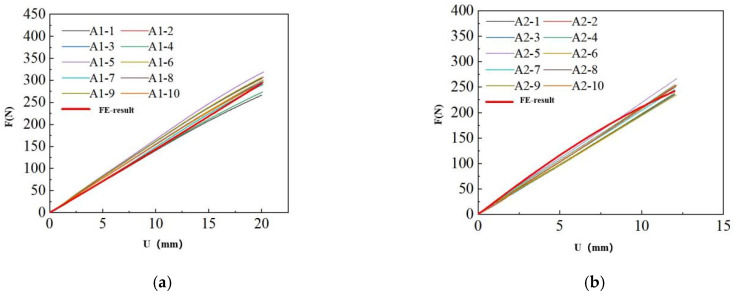

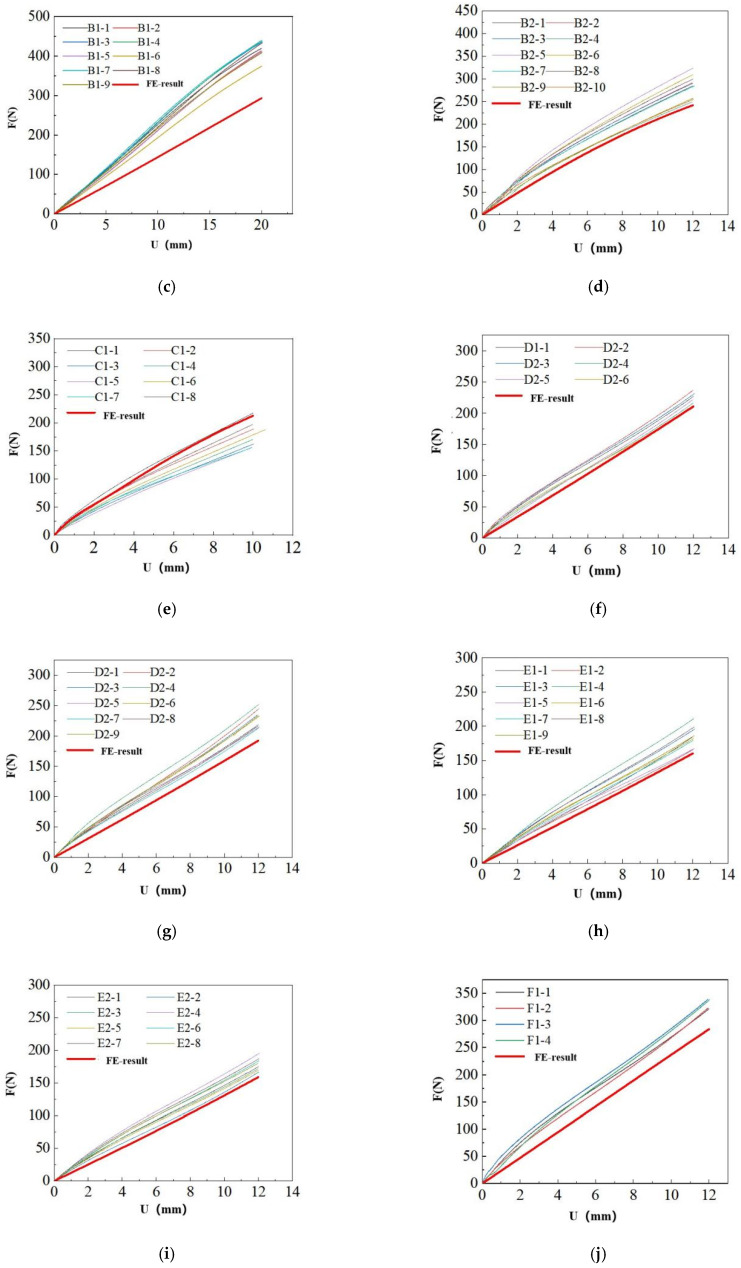
Comparison of shear test results and finite element analysis results for various SSAs: (**a**) Fitting results of the shear force–displacement curve of A1. (**b**) Fitting results of the shear force-displacement curve of A2. (**c**) Fitting results of the shear force–displacement curve of B1. (**d**) Fitting results of the shear force–displacement curve of B2. (**e**) Fitting results of the shear force–displacement curve of C1. (**f**) Fitting results of the shear force–displacement curve of D1. (**g**) Fitting results of the shear force–displacement curve of D2. (**h**) Fitting results of the shear force–displacement curve of E1. (**i**) Fitting results of the shear force–displacement curve of E2. (**j**) Fitting results of the shear force–displacement curve of F1.

In order to further verify the accuracy of the constitutive relation described in this paper, a finite element model is established using the constitutive relation parameters obtained in Section 4. This model is then compared with the shear test results presented in Section 2.2. The comparison results for various types of SSAs are shown in Figure 10 and Table 4.

Under the same conditions, the fitting accuracy of the shear test is relatively lower. Based on the changes observed in the two curves, it can be inferred that the fitting results using these constitutive parameters are less accurate than the actual experimental data. This discrepancy arises because, in this study, the constitutive parameters were fitted using tensile test results that meet the 95% lower limit.

According to the statistical results of the fitting curves, the determination coefficients for most fitting curves exceed 0.85, the mean absolute errors are within 100 N, and the area error ratios for most silicone sealants are below 20%. A determination coefficient above 0.85 indicates a strong correlation between the two curves, suggesting that the data obtained from the finite element analysis of most silicone sealants align well with the experimental test data. Taking the area error ratio as the index for fitting accuracy of the curve, the calculation formula for the area error ratio is |S_fitted-S_test|/S_test. According to the results, the area error ratios of several silicone sealants, such as A1, B2, and D1, are within 10%, indicating that the fitting accuracy exceeds 90%. This suggests that the tensile constitutive models and parameters of these silicone sealants remain applicable in shear tests. However, the area error ratios for other silicone sealants, including A2, C1, D2, E1, E2, and F1, range from 10% to 20%, indicating that their fitting accuracy falls between 80% and 90%. Although the fitting accuracy of these silicone sealants is relatively lower, the results are still reasonably close, suggesting that the tensile constitutive model parameters for these silicone sealants are less applicable to shear tests. According to the fitting curves and statistical results, there is a significant gap in the performance of the B1 silicone sealant. The coefficient of determination between the two is only 0.76, indicating a weak correlation between the fitting curve and the experimental curve. This suggests that the tensile constitutive model parameters for B1 silicone sealant are less applicable to shear tests. However, the area error ratio indicates that the fitting accuracy remains above 70%, implying that this constitutive model is still suitable for the B1 silicone sealant. The poor correlation of the fitted curve may be attributed to the selection of tensile test parameters.

## 6. Conclusions

This paper aims at the issues of limited universality and low accuracy associated with existing phenomenological hyperelastic constitutive relations when applied to curtain wall SSAs. We selected ten types of typical SSAs and conducted tensile tests, shear tests, and finite element numerical analyses. Based on this research, we reached the following conclusions:

(1) There are significant differences in the tensile mechanical properties of various types of SSAs, primarily evident in the failure modes observed during the failure stage. Notably, there is no clear correlation between the tensile and shear mechanical properties of SSAs and their hardness values.
(2) The existing phenomenological hyperelastic constitutive models have limited applicability to SSAs used in glass curtain walls. They particularly struggle to encompass all types, especially those SSAs with significant deformation capabilities. The integral hyperelastic constitutive model proposed in this paper demonstrates improved applicability for SSAs experiencing large deformations and can more accurately represent their tensile and shear properties.
(3) It is feasible to utilize the load–displacement curves of existing I-shaped tensile specimens of SSAs. By employing a parameter optimization algorithm based on the hyperelastic constitutive relation using the AE algorithm, we can invert the constitutive model and its parameters for the silicone structural sealant. This method can further obtain the mechanical property parameters necessary for refined finite element analysis while still meeting the testing requirements of existing specifications.

## Figures and Tables

**Table 1:** The phenomenological model and its strain energy function.

Model	Strain Energy Function	Characteristics
Polynomial Model	$U=\sum_{i+j=1}^{N}C_{ij}{\left(\bar{I_{1}}-3\right)}^{i}{\left(\bar{I_{2}}-3\right)}^{j}+\sum_{i=1}^{N}\frac{1}{D_{i}}{\left(J_{el}-1\right)}^{2i}$	The higher the order of the model, the more precise it becomes; however, determining the parameters also becomes increasingly challenging.
Reduced Polynomial Model	$U=\sum_{i=1}^{N}C_{i0}{\left(\bar{I_{1}}-3\right)}^{i}+\sum_{i=1}^{N}\frac{1}{D_{i}}{\left(J_{el}-1\right)}^{2i}$	The number of calibration parameters has been reduced, making it easier to use; however, this results in slightly lower accuracy.
Mooney–Rivlin	$U=C_{10}\left(\bar{I_{1}}-3\right)+C_{01}\left(\bar{I_{2}}-3\right)+\frac{1}{D_{1}}{\left(J_{el}-1\right)}^{2}$	It is the most common constitutive model and is applicable for medium- to low-strain scenarios.
Neo-Hookean	$U=C_{10}\left(\bar{I_{1}}-3\right)+\frac{1}{D_{1}}{\left(J_{el}-1\right)}^{2}$	It has a simple shape, but the curve is relatively stable, making it suitable for small strains.
Yeoh	$U=\sum_{i=1}^{3}C_{i0}{\left(\bar{I_{1}}-3\right)}^{i}+\sum_{i=1}^{3}\frac{1}{D_{i}}{\left(J_{el}-1\right)}^{2i}$	It has a wide range of applications, accommodates large deformations effectively, and exhibits an S-shaped curve.
Odgen	$U=\sum_{i=1}^{N}\frac{2\mu_{i}}{\alpha_{i}^{2}}\left({\bar{\lambda }}_{1}^{\alpha_{i}}+{\bar{\lambda }}_{2}^{\alpha_{i}}+{\bar{\lambda }}_{3}^{\alpha_{i}}-3\right)+\sum_{i=1}^{N}\frac{1}{D_{i}}{\left(J_{el}-1\right)}^{2i}$	It demonstrates a relatively high fitting accuracy; however, it performs poorly when fitting individual data points.
Arruda–Boyce	$U=\mu \sum_{i=1}^{5}\frac{C_{i}}{\lambda_{m}^{2i-2}}\left({\bar{I}}_{1}^{i}-3^{i}\right)+\frac{1}{D}\left(\frac{J_{el}^{2}-1}{2}-\ln(J_{el})\right)$	The fitting accuracy is relatively low, and it is applicable only when there is limited data.
Van der Waals	$U=\mu \left\{-\left(\lambda_{m}^{2}-3\right)\left[\ln\left(1-\eta \right)+\eta \right]-\frac{2}{3}a{\left(\frac{\tilde{I}-3}{2}\right)}^{\frac{3}{2}}\right\}$	The fitting accuracy is relatively low and is applicable only when there is limited data.

**Table 2:** Labels and applicable scopes of ten typical types of silicone structural sealants.

Label	Manufacturer	Model	Hardness Value	Scope of Application
A-1	HSK, Shanghai, China	995	31.9	Filling gaps around doors and windows, as well as indoor caulking and bonding, etc.
A-2	HSK, Shanghai, China	8800	37.7	Large-scale glass curtain walls, stone curtain walls, metal curtain walls, etc.
B-1	WJDH, Shandong, China	8800	34.8	Door and window caulking, small-scale curtain wall structures, etc.
B-2	WJDH, Shandong, China	9800	44.8	Large-scale curtain walls, sunrooms, etc.
C-1	DHRT, Guangzhou, China	8800	40.2	Large-scale curtain walls, sunrooms, etc.
D-1	BYSS, Guangzhou, China	621	42.6	Door and window caulking, curtain wall structures, etc.
D-2	BYSS, Guangzhou, China	521	42.1	Bonding and sealing of glass, base materials, stones, and curtain wall structures, etc.
E-1	DKN, USA	SJ268	37.4	Glass, stone, metal curtain walls, etc.
E-2	DKN, USA	995	36.3	Glass, stone, metal curtain walls, etc.
F-1	GB, Sichuan, China	999	48.9	Doors, windows, sunrooms, curtain wall structures, etc.

**Table 3:** Inversion results and errors of constitutive relation parameters for ten types of SSAs.

Silicone Sealant	*C*_10_	*C*_20_	*C*_30_	*C*_40_	*C*_50_	*C*_60_	Error
A1	0.24	0.7	0.085	1.00	−85	1.20	79
A2	0.37	0.8	0.055	0.85	−2000	2.20	28
B1	0.61	0.8	0.055	0.90	−12,000	2.20	42
B2	0.305	0.9	0.075	0.95	−200	0.95	38
C1	0.39	1.2	0.078	0.83	−50	0.80	81
D1	0.66	4.0	0.080	0.01	−2000	0.65	451
D2	0.35	1.5	0.060	0.02	−20	0.45	290
E1	0.23	1.0	0.055	0.05	−50	0.60	43
E2	2.30	300.0	0.145	0.50	−100	0.10	593
F1	0.45	1.2	0.070	0.20	−50	0.45	195

**Table 4:** Fitting results of ten silicone adhesive shear tests.

Silicone Sealant	Maximum Absolute Error/N	Mean Absolute Error/N	Mean-Squared Error	Area Error Ratio	Coefficient of Determination
A1	11	7	60	4.60%	0.99
A2	20	13	217	11.20%	0.95
B1	76	47	2880	24.47%	0.76
B2	15	10	113	7.25%	0.98
C1	24	10	190	10.43%	0.93
D1	11	8	68	7.15%	0.98
D2	24	15	264	14.25%	0.92
E1	24	17	327	17.62%	0.88
E2	22	18	374	19.10%	0.85
F1	40	25	710	15.30%	0.91

## Data Availability

The original contributions presented in this study are included in the article. Further inquiries can be directed to the corresponding author.
